# Transjugular Intrahepatic Portosystemic Shunt in Chronic Portal Vein Thrombosis—From Routine Recommendations to Demanding Scenarios

**DOI:** 10.3390/diagnostics12123100

**Published:** 2022-12-09

**Authors:** Sasidharan Rajesh, Shobhit Singh, Cyriac Abby Philips

**Affiliations:** 1 Interventional Hepatobiliary Radiology, The Liver Institute, Center of Excellence in GI Sciences, Rajagiri Hospital, Chunangamvely, Aluva 683112, Kerala, India; 2 Clinical and Translational Hepatology & Monarch Liver Laboratory, The Liver Institute, Center for Excellence in Gastrointestinal Sciences, Rajagiri Hospital, Aluva 683112, Kerala, India

**Keywords:** TIPS, EHPVO, anticoagulation, transhepatic, cavernoma, collateral, endovascular, cirrhosis, PVT, portosystemic shunt

## Abstract

Portal vein thrombosis (PVT), particularly the presence of portal cavernoma, was traditionally considered a relative contraindication for transjugular intrahepatic portosystemic shunting (TIPS) due to the technical difficulties in accessing and maneuvering the portal vein and avoiding the high risk for bleeding periportal collaterals. However, the last decade has seen a surge in the number of studies—mostly case reports and small series of patients—demonstrating that TIPS is not only technically feasible in the vast majority of these patients but also provides effective and long-term control of symptoms associated with portal hypertension in cases refractory to the standard line of therapy. The present article aims to provide a concise but exhaustive overview of the role and the standard and technically difficult TIPS placement scenarios in patients with chronic non-malignant PVT and with and without underlying liver disease. The review is strategically punctuated by exemplary instances from the authors’ experience.

## 1. Introduction

Portal vein thrombosis (PVT) refers to the obstruction of the portal venous blood flow due to a thrombus in the lumen of the vein. The splenic and superior mesenteric veins can also be variably involved in this context. PVT is estimated to affect 1–2% of the world population, and its prevalence continues to increase [1,2]. The presence of an underlying prothrombotic state, local abdominal inflammation, malignancy, cirrhosis, and iatrogenic causes have all been implicated in etiopathogenesis [1,2]. Depending on the extent of the thrombus, the rapidity with which it develops, and the status of the underlying liver, PVT can have different clinical consequences. Acute extensive thrombosis can present as fever, abdominal pain in the presence or absence of variceal bleeding, and ascites. Contrarily, PVT may be entirely asymptomatic, even in the acute stage. It may be detected as an incidental finding from imaging long after progressing to chronicity and cavernoma formation. In the chronic stage, the commonest clinical presentations in a non-cirrhotic patient are well-tolerated episodes of variceal bleeding often accompanied by splenomegaly or anemia. Additionally, in pediatric cases, associated growth retardation is notable [3]. Ascites, symptomatic portal cavernoma cholangiopathy (previously—portal biliopathy), and intestinal ischemia are less common. Patients with cirrhosis and chronic PVT with or without cavernoma formation can present with difficult-to-treat ascites and recurrent or difficult-to-control variceal bleeding. Importantly, PVT is an additional challenge when deteriorating liver functions necessitate a liver transplant (LT). Although modifications in surgical approaches have allowed for successful transplants in this scenario, patients with PVT have poor survival after transplant compared with patients with a patent portal vein [4]. This exhaustive review aims to describe interventional strategies for patients with chronic PVT with and without cirrhosis, with special consideration for transjugular intrahepatic portosystemic shunt (TIPS) placement. Further, we discuss technical challenges associated with the TIPS procedure in unique patient situations and provide expert solutions.

## 2. Interventional Strategies in Chronic PVT

Variceal bleeding in chronic non-cirrhotic PVT can be successfully managed by vasoactive drugs and endoscopic band ligation, which have low morbidity but requires repeated visits and adherence to long-term surveillance protocols or by portosystemic shunt surgery. The latter provides good control of bleeding, helps improve growth retardation in children, ameliorates hypersplenism, and protects against the future development of gastric and ectopic varices. It also helps control the worsening of portal cavernoma cholangiopathy but could be associated with surgical mortality. It is sometimes not feasible due to the non-availability of a satisfactory blood vessel that precludes vascular reconstruction [5]. In 8–12% of cases, endoscopic therapy fails to control the acute episode of variceal bleeding [3]. In patients who are not surgical candidates, endovascular therapies such as portal vein recanalization (PVR), TIPS, and percutaneous variceal embolization are effective options for arresting the bleeding [6].

In patients with chronic PVT due to cirrhosis, treatment options and therapy goals differ from the non-cirrhotic group, which includes not only the management of symptomatic portal hypertension but also an improved chance of LT candidacy or transplant-free survival. Typical indications for endovascular intervention include recurrent variceal hemorrhage, refractory ascites, and hepatic hydrothorax. Asymptomatic patients who do not need LT can be observed to develop complications requiring interventions [7,8].

## 3. Endovascular Therapies

Since chronic non-cirrhotic PVT leads to prehepatic portal hypertension, and intrahepatic resistance is often relatively normal, several studies have explored the role of PVR without placing a TIPS stent in these patients [9]. This approach restores physiological blood inflow to the liver and prevents complications such as portosystemic encephalopathy, hepatic ischemia, and cardiac failure associated with TIPS. However, ensuring the patency of intrahepatic branches of the portal vein and splenic and superior mesenteric veins and ruling out advanced fibrosis is a prerequisite to PVR to prevent technical and clinical failure and early stent thrombosis. In well-selected patients, technical success rates of 87–100% have been reported, with 5-year primary patency rates of up to 73% [9]. Recent studies have also demonstrated an improvement in sarcopenia and a reduction in splenic volumes after PVR [9]. Therefore, before contemplating any endovascular therapy in patients with chronic non-cirrhotic PVT, careful evaluation, which includes mandatory cross-sectional imaging, is paramount to assess whether the patient could be a candidate for PVR.

Percutaneous variceal embolization, although effective in the acute setting with 70–90% success rates, does not take care of the underlying portal hypertension, and a high incidence of re-bleeding (37–65%) remains a concern [10,11,12]. Moreover, embolization of large shunts during variceal embolization can lead to the development of new-onset ascites in a significant proportion of patients.

While TIPS has become an established treatment modality in managing cirrhotic patients with variceal bleeding or difficult-to-treat ascites, placement of the TIPS stent in patients with chronic PVT and portal cavernoma formation is technically challenging and, until some years ago, was considered a contraindication.

## 4. Technical Challenges to TIPS and Modifications in Approach

TIPS involves creating an artificial conduit between the hepatic and portal veins to decrease the portal pressure and resolve the associated complications of portal hypertension. The procedure has traditionally been performed under fluoroscopic guidance with or without wedge portography or carbon-dioxide angiography to delineate the portal venous branches [13]. Both the presence of chronic PVT and portal cavernoma pose unique challenges to the placement of the TIPS stent. Occluded veins are difficult to demarcate on portography, and the advancement of the guidewire and catheters through the occluded segments is often tested. Moreover, local or systemic thrombolysis is often performed simultaneously with recanalization in this setting, increasing the risk of bleeding complications. Accordingly, initial TIPS studies in PVT reported only partial success and often had to resort to unconventional means to achieve the outcome [14]. With the advent of real-time ultrasound guidance (either transabdominal or intravascular) to visualize the occluded native portal vein during TIPS and multiple modifications to the TIPS approach, recent studies have reported high technical success rates [15,16]. In addition, the universal usage of expanded-polytetrafluoroethylene stent grafts for TIPS has resulted in better long-term patency rates. Various modifications in the approach to TIPS in chronic PVT have been detailed below:

### 4.1. Transjugular Approach

**Figure 1:**
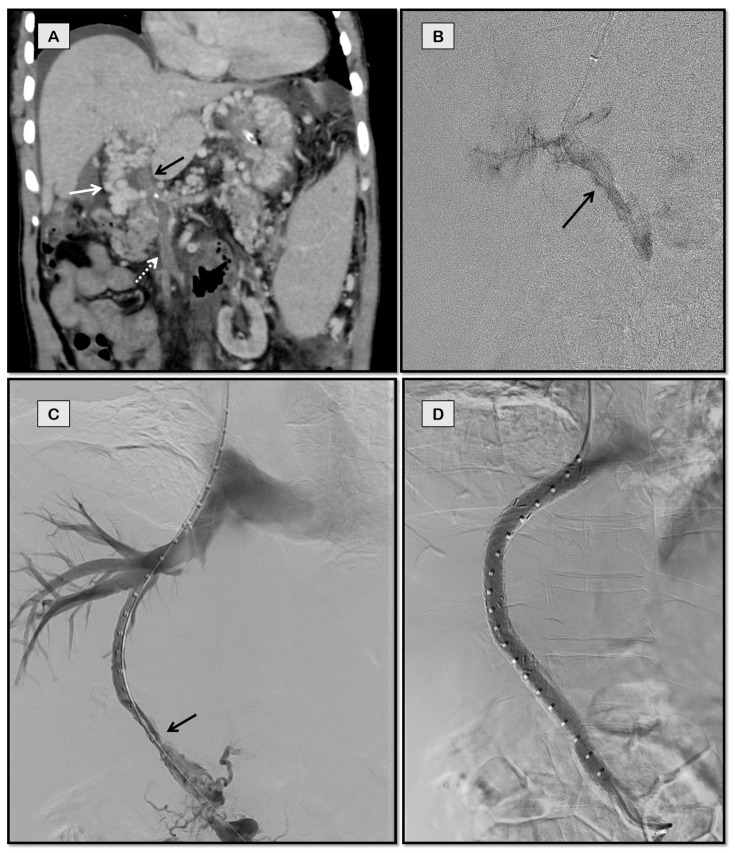
TIPS in a 61-year-old man with polycythemia vera and chronic porto-mesenteric vein thrombosis who presented with recurrent variceal bleed refractory to endoscopic therapy. Patient was not a surgical candidate due to multiple comorbidities. Coronal-oblique image (**A**) from the computed tomographic (CT) scan of the patient showing chronic thrombosis of the portal vein (black arrow) and superior mesenteric vein (dashed arrow) with cavernoma formation (white arrow). Fluoroscopic spot image (**B**) shows the thrombosed portal vein (arrow) accessed from jugular approach. Post balloon maceration and thromboaspiration, there is partial recanalization of the portal and superior mesenteric vein (**C**). Post-stenting (**D**) image shows brisk flow through the stent with absence of collateralization.

**Figure 2:**
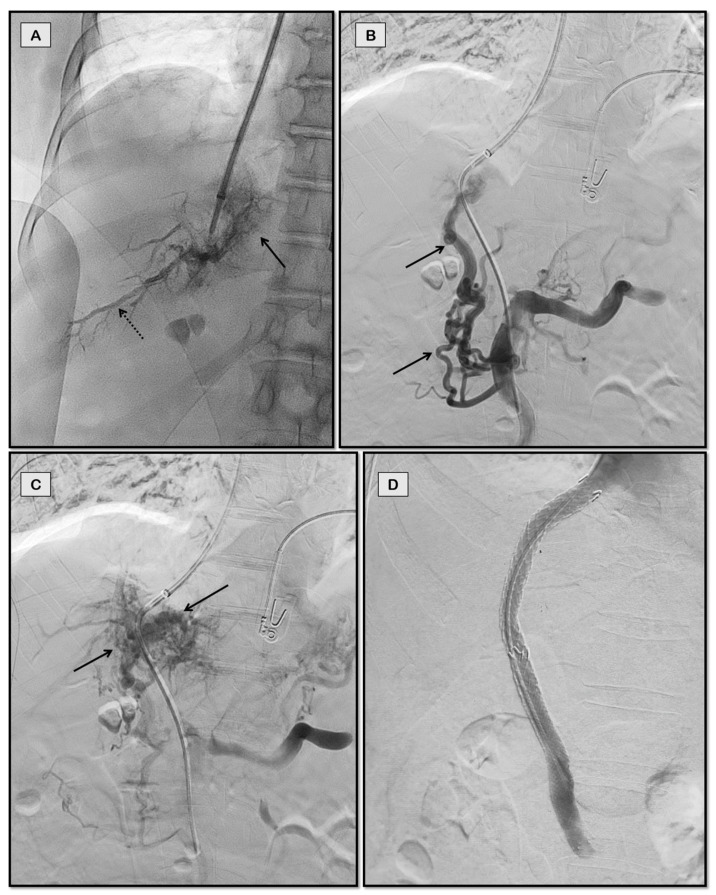
TIPS for recurrent variceal bleeding in a 63-year-old woman with chronic non-cirrhotic portal vein thrombosis. Fluoroscopic spot image (**A**) shows the partially thrombosed posterior segmental branch of right portal vein (dashed arrow) accessed through jugular approach. Abnormal parenchymal blush is also noted (solid arrow) signifying poor inflow to liver. Superior mesenteric vein angiogram (**B**,**C**) showed completely thrombosed portal vein with multiple periportal collaterals (arrows in **B**) and portal cavernoma (arrows in **C**). Brisk flow was noted post-stenting (**D**).

**Figure 3:**
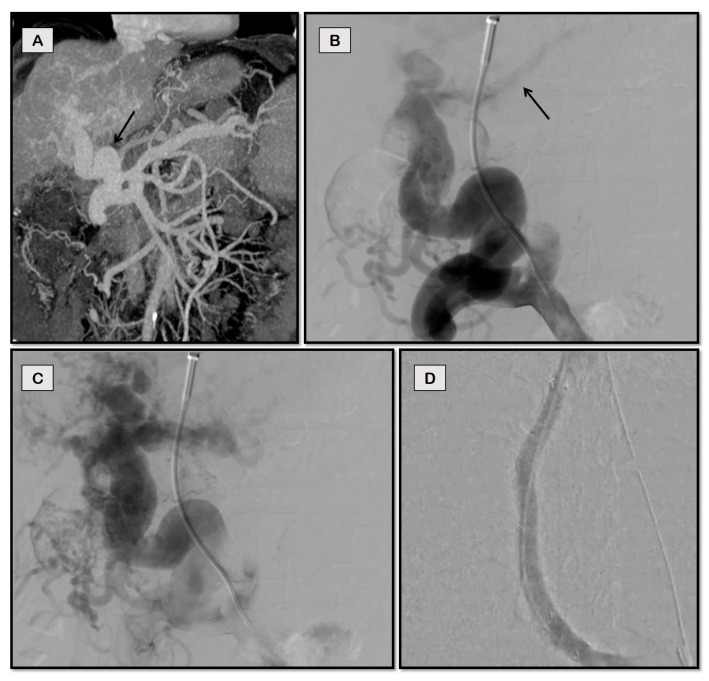
TIPS for refractory ascites in a 53-year-old man with cirrhosis and chronic portal vein thrombosis due to non-alcoholic steatohepatitis (NASH). Coronal-oblique CT image (**A**) shows a large periportal collateral (arrow) replacing the native main portal vein. The main portal vein was accessed through middle hepatic vein via partially patent left portal vein (faintly opacified on **B**, arrow) from the jugular approach. Image (**C**) shows the portal cavernoma. Optimal flow of contrast was noted post-stenting (**D**) with disappearance of collaterals.

A standard transjugular approach with real-time transabdominal or intravascular ultrasound guidance remains one of the preferred approaches to TIPS in chronic PVT (Figure 1, Figure 2 and Figure 3). Simultaneous ultrasound guidance allows the operator to access the thrombosed native portal vein. Use of this route not only reduces the procedure time and associated risks of prolonged radiation exposure but avoids several other potentially life-threatening complications of TIPS such as capsular transgression and hemoperitoneum, inadvertent puncture of an artery or periportal collateral vessels, biliary injury, and access site complications (in cases of transhepatic or trans-splenic approach) [17]. The need for an extra set of expert hands for ultrasound guidance and unfavorable body habitus in some patients remains a drawback. Moreover, the thrombosed or fibrotic intrahepatic branch of the native portal vein should be visible on the ultrasound for this approach to work. The fluoroscopy-guided puncture of the non-visualized fibrotic portal vein from the transjugular approach has found limited technical success.

### 4.2. Transhepatic Approach

**Figure 4:**
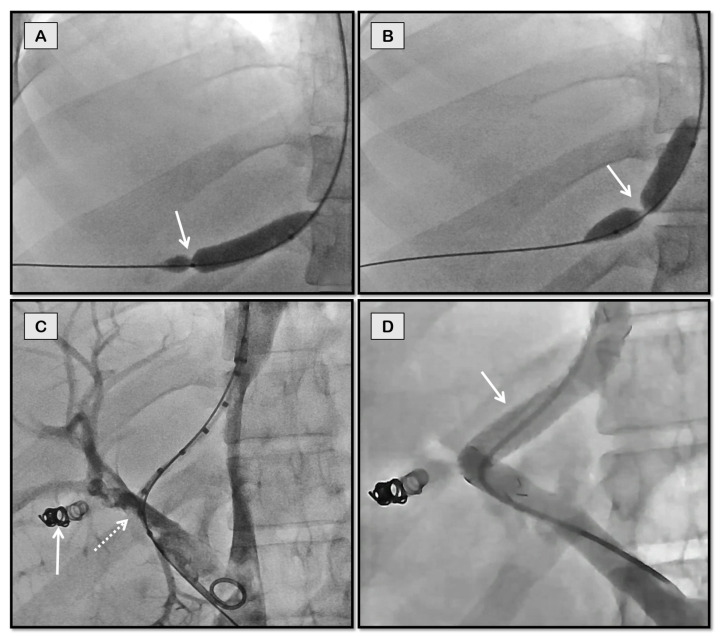
Percutaneous transhepatic access for portosystemic shunting in a 26-year-old man with chronic hepatic and portal vein thrombosis with refractory ascites. Fluoroscopic spot images (**A**,**B**) show through-and-through access obtained between percutaneous transhepatic and jugular puncture sites with a balloon inflated to demonstrate the portal puncture site (arrow in **A**) and IVC puncture site (arrow in **B**). Post balloon maceration and thromboaspiration (**C**), there is partial recanalization of the portal vein (dashed arrow). Coils used to close the percutaneous tract can also be seen in this image (solid arrow). Good flow across the stent was noted on the completion angiogram (arrow in **D**).

Initial attempts at TIPS in patients with portal vein thrombosis utilized the transhepatic approach [14]. Briefly, a micropuncture set is used to access the thrombosed portal vein under fluoroscopic or ultrasound guidance. After balloon dilatation and thrombus aspiration from the portal vein, an inflated contrast-filled balloon or vascular snare is placed, which can be used as a target to puncture the portal vein from the transjugular approach. The rest of the procedure is completed from the jugular side in the standard fashion. The transhepatic tract is plugged with gel foam, coils, or glue. In patients with concomitant chronic thrombosis of hepatic veins (chronic Budd–Chiari Syndrome), this approach can be modified by simultaneously puncturing the thrombosed portal vein and the inferior vena cava (IVC) and snaring the guidewire from IVC through the jugular route (Figure 4).

The tract between the portal vein and IVC is then balloon dilated, and the portal vein is accessed from the jugular approach to stent deployment. The advantage of the transhepatic approach is that accessing the portal system via this shorter route through the liver under image guidance is considered relatively easier compared to the longer transjugular route wherein a longer needle has to be manipulated through the metallic cannula. However, concern for access site complications remains, especially in patients with coagulopathy and ascites. With the advent of intravascular ultrasound and the trans-splenic approach, the transhepatic route is less commonly utilized.

### 4.3. Trans-Splenic Approach

**Figure 5:**
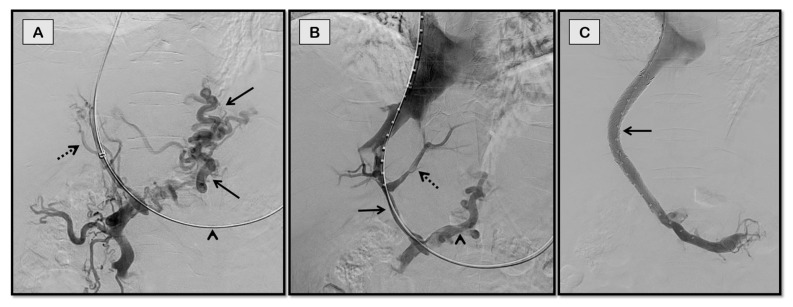
Trans-splenic access for TIPS for recurrent variceal bleeding in a 72-year-old man with chronic portal vein thrombosis secondary to NASH-cirrhosis. Fluoroscopic spot image (**A**) of splenoportal angiogram obtained after gaining through-and-through access between jugular and trans-splenic puncture sites (arrowhead) shows completely thrombosed portal vein with periportal collaterals (dashed arrow) and tortuous left gastric vein giving rise to esophageal varices (solid arrows). Post balloon maceration and thromboaspiration (**B**), there is partial recanalization of the main portal vein (solid arrow) and its intrahepatic branches (dashed arrow), but the persistent filling of the left gastric vein (arrowhead). Image (**C**) shows optimal contrast flow through the TIPS stent with the disappearance of collaterals.

A significant proportion of patients with portal cavernoma do not have a visible native portal vein on imaging. Thus, alternative routes had to be devised for the placement of TIPS. The trans-splenic approach remains the mainstay for such patients. Originally described in the late 1980s, the trans-splenic route was traditionally associated with high rates of hemorrhagic complications [18]. However, with improved imaging and real-time ultrasound guidance, complication rates directly attributable to the splenic puncture became low [19]. The trans-splenic route is considered ideal when there is a large patent splenic vein with one of its branches entering the splenic parenchyma in a direct anatomic or straight line [20]. This intraparenchymal branch is punctured under ultrasound guidance using a micropuncture set. A guidewire and catheter combination are then used to probe and access the fibrotic portal vein via an antegrade approach. The technique requires repeated probing with multiple different catheters at the splenoportal confluence to succeed. After recanalizing the portal vein, a contrast-filled balloon or vascular snare can be placed within the third-order portal vein, which can be targeted via the transjugular route using fluoroscopic triangulation to get the guidewire into the portal vein from the jugular side. This guidewire is snared from the trans-splenic access route to create through-and-through access (Figure 5).

**Figure 6:**
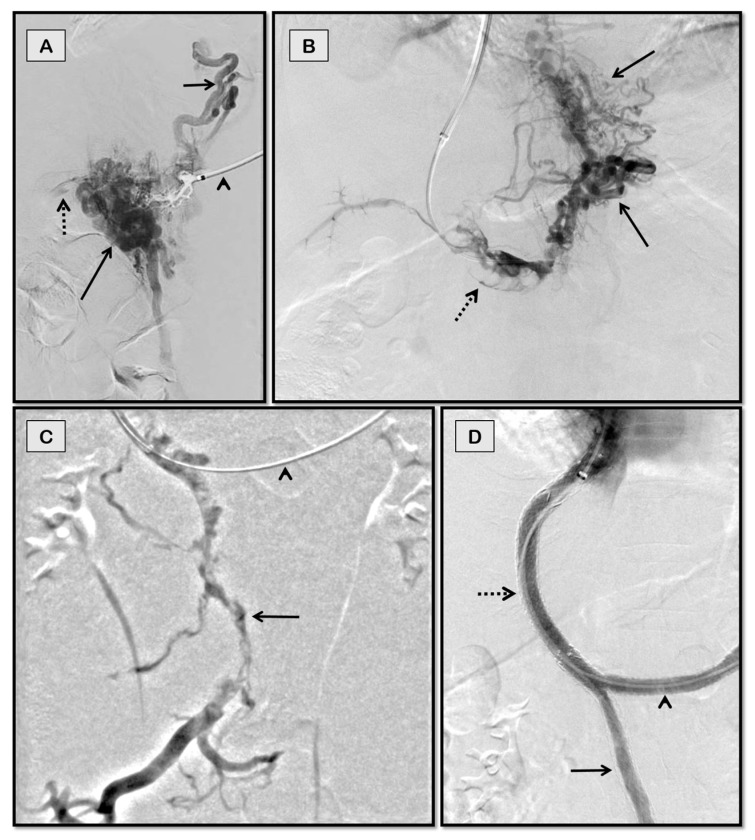
Trans-splenic TIPS for recurrent variceal bleeding in a 63-year-old man with non-cirrhotic chronic porto-mesenteric and splenic vein thrombosis. Fluoroscopic spot image (**A**) of angiogram obtained through trans-splenic route (arrowhead) showing tortuous short gastric vein (short arrow) and perisplenic collaterals (long arrow). Splenic vein is faintly opacified (dashed arrow). Venogram images (**B**,**C**) captured after obtaining transjugular access through splenic route (arrowhead in **C**) showing completely thrombosed portal and superior mesenteric veins (dashed arrow in (**B**) and solid arrow in (**C**), respectively) with varices (solid arrows in **B**). Image (**D**) depicts bifurcated Y-shaped stents in splenoportal axis showing brisk flow with disappearance of collaterals.

The rest of the procedure is completed via neck using a standard technique. The trans-splenic access site is plugged in to avoid bleeding complications. This approach can also be used in patients with partial thrombosis of the splenic vein at the splenoportal confluence in whom accessing the splenic vein from the transjugular route is difficult (Figure 6).

### 4.4. Transmesenteric Approach

Recent studies have described the percutaneous transmesenteric approach to access the portal vein via the antegrade route [21,22]. For patients in whom primary transjugular, transhepatic, or trans-splenic approaches fail due to chronically thrombosed splenic veins with non-visualization of intrahepatic native portal vein branches or those who have undergone splenectomy, this route can be utilized. A 21-G needle is used to access the superior or inferior mesenteric vein via the transabdominal route under ultrasound guidance. A guidewire and catheter combination are then used to catheterize the thrombosed portal vein. Post recanalization of the portal vein, a snare is placed within it, which can be used as a target from the transjugular side for access. This guidewire inserted from the jugular route is snared from the percutaneous mesenteric vein entry site to establish Archimedean access. The procedure is thereafter completed via the jugular route using the standard technique.

### 4.5. Collateral Vein Stenting

**Figure 7:**
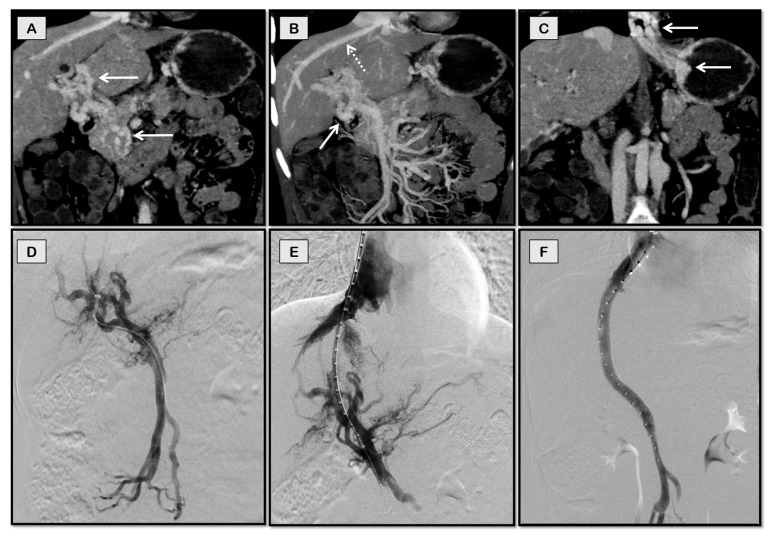
TIPS through a collateral vein for recurrent variceal bleeding in a 17-year-old girl with chronic non-cirrhotic portal vein thrombosis. She had undergone a splenectomy at the age of 6 years and was not a candidate for Rex shunt. Coronal-oblique CT images (**A**–**C**) showing portal cavernoma (arrows in **A**), dominant collateral vein (dashed arrow in **B**) in the same plane as middle hepatic vein (solid arrow in **B**), and esophageal and gastric varices (arrows in **C**). The collateral vein was accessed through a jugular route (**D**) and stented (**E**) with flow restoration and disappearance of collaterals (**F**).

**Figure 8:**
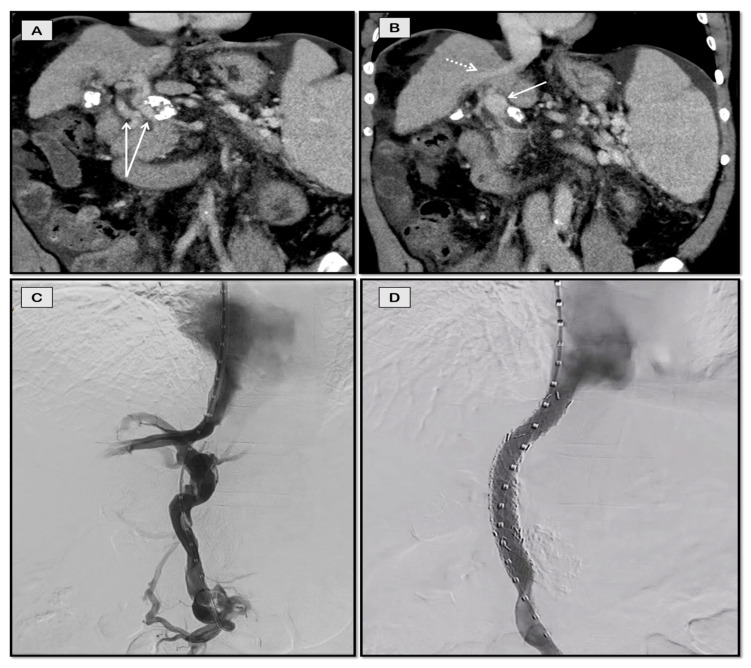
TIPS through collateral vein for refractory ascites in a 59-year-old man with chronic portal vein thrombosis secondary to NASH-cirrhosis. Coronal-oblique CT images (**A**,**B**) showing portal cavernoma (arrows in **A**) and dominant collateral vein (solid arrow in **B**) in the same plane as middle hepatic vein (dashed arrow in **B**). Collateral vein was accessed through jugular route (**C**) and stented with restoration of flow (**D**).

Large, relatively less tortuous periportal collateral veins communicating with the superior mesenteric vein or splenic vein inferiorly can sometimes be used to create TIPS in patients who had a failure of TIPS via other approaches. These collaterals and the possible route through which they can be accessed via any of the three hepatic veins can be evaluated by preprocedural imaging (Figure 7 and Figure 8).

**Figure 9:**
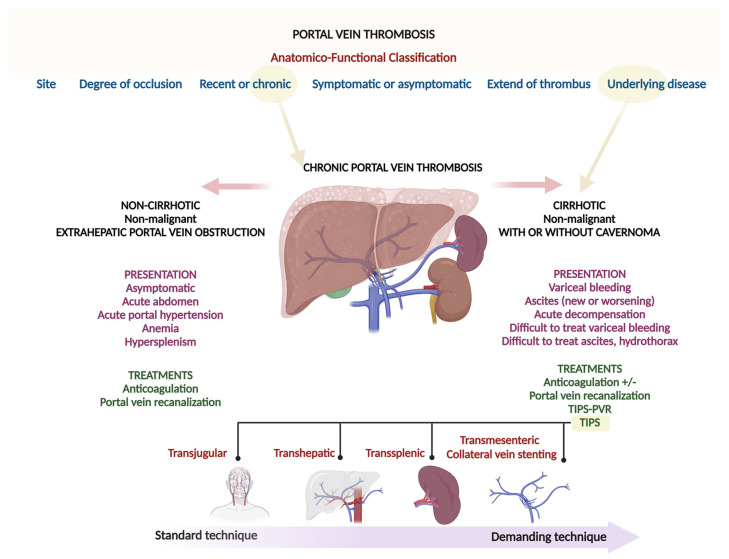
Summary infographics.

However, accessing a suitable collateral vein is often difficult and risks extrahepatic portal vein laceration and hemoperitoneum. An infographic summary of chronic PVT and its pertinent clinical and therapeutic features is shown in Figure 9.

## 5. TIPS—Technical Success, Complications, and Long-Term Patency

Reports of TIPS in portal vein thrombosis have been markedly heterogeneous. A significant proportion of these studies have included both cirrhotic and non-cirrhotic patients in the cohort with different degrees of portal vein thrombosis of varying chronicity. Moreover, the presence or absence of portal cavernoma has not been universally mentioned. The approaches used for TIPS have also been diverse.

In one of the earlier studies on TIPS in chronic portal vein thrombosis, Senzolo et al. reported the outcomes of endovascular treatment in a heterogeneous cohort of 28 patients [15]. The study group included both cirrhotic and non-cirrhotic patients. The authors found that 23 of 28 patients had complete portal vein thrombosis, and 9 of 23 had a cavernous transformation. They used a transjugular approach in all patients and could achieve a technical success rate of 73%. Although multiple instances of capsular and biliary punctures were reported in this study, none had any major sequelae. One patient had an extrahepatic portal vein laceration successfully treated with a covered stent. The TIPS group had significantly lower re-bleeding rates compared to the failed-TIPS group. After a mean follow-up of 18.1 months, primary TIPS patency was maintained in 74% of patients. The authors, for the first time, in a moderately large cohort, showed that portal cavernoma should not be seen as a contraindication to TIPS. However, it increases the technical difficulty of the procedure.

In another study on TIPS in 17 patients with chronic non-cirrhotic PVT using the transjugular approach, a technical success rate of 76.5% was reported [23]. The cumulative 24-month secondary portal vein patency was 69.5%. Two major bleeding complications were reported in this study, both of which were resolved with conservative management.

The percutaneous transhepatic route with balloon or snare assistance for the placement of TIPS in chronic PVT has been described in several recent studies. One of these studies reported outcomes in 18 patients with cirrhosis and chronic PVT [24]. The authors did not use ultrasound guidance to access the thrombosed portal vein. TIPS was technically successful in 14 patients (78% technical success rate). The four failure cases were those in whom there were no detectable intrahepatic portal vein branches on imaging. No major procedure-related complications were observed. During a median follow-up period of 16 months, only one of these patients exhibited features of stent dysfunction and underwent a shunt revision. Other studies (mostly case reports and small case series) have reported that the transhepatic approach is useful in patients in whom the transjugular approach is not feasible or fails, with excellent technical success rates [14,25,26].

In a series of 44 pretransplant cirrhotic patients with complete (100%) or near-complete (95%) chronic PVT, 30% of whom had portal cavernoma, technical success rates of 98% were reported with 5-year portal vein patency rates of 89% [27]. The authors used a combination of various percutaneous approaches (transjugular, transhepatic, as well as trans-splenic) for TIPS in this study. Even better results were reported by authors from the same institute in a group of 11 patients using a trans-splenic approach [20]. Technical success was achieved in all patients without any major complications. All patients had patent TIPS at a median follow-up of 6.4 months. Similar results have been reported in non-cirrhotic patients in a smaller case series of 5 patients, in whom TIPS was technically successful in all patients [16]. At a mean follow-up of 8.2 months, all the TIPS stents were patent. These results are in stark contrast to the studies described above using the transjugular and transhepatic approach in which 22–27% of TIPS placements were unsuccessful and up to 32% of technically successful placements needed revisions [15,23]. Thus, the trans-splenic route could be useful even in patients whose TIPS placement failed from other approaches.

Other studies reporting outcomes of TIPS in pretransplant patients have also been promising. In a retrospective analysis of 35 patients receiving TIPS for chronic obliterative PVT before LT, it was found that TIPS demonstrated efficacy in resolving PVT and simplified the surgical aspects of LT, allowing for end-to-end portal vein anastomoses [28]. Another analysis of 30 patients who underwent TIPS to maintain PV patency while being listed for LT showed that 24 patients (80%) had improvement and/or the resolution of PVT after TIPS placement, with 18 of these (75%) having a complete resolution [29]. All nine patients who underwent LT after shunt placement received end-to-end anastomosis without requiring intraoperative thrombectomy.

Two meta-analyses of TIPS in cirrhotic PVT have shown that the placement of TIPS alone improved PVT owing to improved portal hypertension and restored PV flow [30,31]. Post-TIPS PVT resolution rates of 74% and 78% and encephalopathy rates of 25% and 16% were reported. Transmesenteric TIPS has been recently described in small study cohorts from a single center [21,22]. Excellent technical success rates were reported by the authors without any major access site complications. In patients with significant thrombus burden in the splenic vein and those who have undergone splenectomy, this approach could be a useful addition to the armamentarium of the interventional radiologist.

One of the largest studies on the role of TIPS in chronic non-cirrhotic PVT was reported recently in a group of 39 patients [22]. All the patients had chronic PVT and portal cavernoma with and without complete mesenteric/splenic vein thrombosis. They presented for management of variceal bleeding, abdominal pain, ascites, bowel ischemia, or portal cavernoma cholangiopathy refractory to the standard of care. The authors used a combination of standard transjugular, transhepatic, trans-splenic, and transmesenteric routes to place TIPS. The trans-splenic approach was used in the maximum number of patients (49%). Technical success was achieved in all patients. At 36 months follow-up, the primary patency of the TIPS stent was 63%, while overall patency was 81%. No correlation was found between TIPS thrombosis and the resumption of anticoagulation. Furthermore, 87% of patients with >6 months follow-up exhibited overall improvement in their clinical condition and/or biochemical parameters. Three patients (8%) developed post-TIPS encephalopathy, one of which was refractory and required shunt constraining. None had hepatic decompensation. Three hepatic hematomas were also observed, of which two required active interventions. The study showed that TIPS placement in chronic non-cirrhotic PVT is associated with significant clinical improvement with an acceptable safety profile (Table 1).

## 6. The Role of Anticoagulation

In acute PVT, experts recommend a minimum of six months of anticoagulation therapy and a longer duration when the thrombus extends to the mesenteric veins, and life-long in the presence of an underlying confirmed prothrombotic state [32]. There are no recommendations for anticoagulation in chronic PVT in cirrhosis, and it remains an individualized decision. Early identification and initiation of anticoagulation therapy increased portal vein recanalization and reduced thrombus progression [33]. Delayed PVT diagnosis and advanced liver disease, as reflected by the model for end-stage liver disease and Child–Pugh scores, are associated with low recanalization rates [34]. TIPS placement before anticoagulation resulted in better thrombus stabilization or complete recanalization in persons with cirrhosis and chronic PVT. Low molecular weight heparin was found to have superior recanalization rates than oral vitamin K antagonists, and data on newer oral anticoagulants in this special scenario are lacking [35]. Chronic PVT, partial, or complete cavernomatous transformation of the portal vein, are exclusions to start anticoagulation, and if technically feasible, early consideration for TIPS is preferred. Anticoagulation after the TIPS procedure in cirrhosis and chronic PVT is not routinely recommended except in cases where a confirmed, underlying prothrombotic condition is diagnosed.

## 7. Conclusions

Multiple technical modifications to the standard TIPS approach in patients with chronic PVT have been reported in recent years. Given the high rates of technical and clinical success described in these studies, the presence of portal cavernoma should not be considered a contraindication to TIPS. However, the potential risks involved with the procedure and the relatively inferior long-term patency rates mandate that the procedure be offered only to patients with severe recurrent complications of portal hypertension or portal cavernoma cholangiopathy that are not responding to standard medical and endoscopic therapy and who are not candidates for shunt surgery or portal vein recanalization. In patients with cirrhosis, TIPS can be utilized to manage the complications of portal hypertension and improve LT candidacy. Referral to centers with substantial experience in TIPS procedures would be desirable to optimize the clinical outcomes. Long-term clinical effects of TIPS in young patients with non-cirrhotic chronic PVT and associated cardiomyopathy need additional studies. In addition, the role of adjuvant anticoagulation in augmenting TIPS patency merits further investigation.

## Figures and Tables

**Table 1:** Summary of major studies on transjugular intrahepatic portosystemic shunt placement in chronic portal vein thrombosis.

Study	Patients	Cirrhotic Non-cirrhotic	Cavernoma	Technical Success (%)	Mean Follow-Up (Months)	Patency (%)	Major Complication	Comments
Senzolo 2006 [15]	28	Both	9/28	73	18.1	74	1/28	The first large cohort study to show cavernoma is not a contraindication for TIPS Technically difficult Capsular and biliary punctures Extrahepatic portal vein laceration No fatal adverse events
Klinger 2018 [23]	17	Non-cirrhotic	15/17	76.5	22.8	44.7	2/17	Capsule rupture, intraperitoneal bleeding Liver hematoma Complex and difficult procedures, which only specialized centers with high experience should attempt
Chen 2015 [24]	18	Cirrhotic	18/18	78	16	92.8	0/18	The technical difficulty leading to open portosystemic shunt placement in four Two deaths reported were not related to the procedure
Salem 2015 [27]	44	Cirrhotic	13/44	98	60	89	0/44	TIPS-assisted recanalization led to the complete resolution of portal vein thrombus in 76% without anticoagulation Transplant-free survival was 82% at five years
Talwar 2021 [28]	35	Cirrhotic	17/35	100	-	69	7/35	TIPS-assisted recanalization is effective in resolving portal vein thrombosis Allowed for end-to-end portal vein anastomoses TIPS-assisted recanalization is a viable treatment option for chronic obliterative portal vein thrombosis with or without cavernoma that eases technical aspects during liver transplantation
Knight 2021 [22]	39	Non-cirrhotic	39/39	100	36	63	3/39	TIPS in chronic, non-cirrhotic extrahepatic portal vein obstruction with cavernomas and mesenteric venous thrombosis is technically feasible TIPS does not adversely affect liver function in this technically difficult-to-intervene group of patients

## Data Availability

Not applicable.
